# Retraction Note: Cental macular thickness in patients with type 2 diabetes mellitus without clinical retinopathy

**DOI:** 10.1186/s12886-015-0114-z

**Published:** 2015-11-16

**Authors:** Mehmet Demir, Ersin Oba, Burcu Dirim, Erhan Ozdal, Efe Can

**Affiliations:** Sisli Etfal Training and Research Hospital, Eye Clinic, Karayolları Mah, Abdi ipekci bulvarı. N0:32 Avrupa tem konutları 28. Blok. Daire:14. 34250 GOP, Sisli, Istanbul 34400 Turkey

The corresponding author submitted this article [1] to the *Journal of Ophthalmology* although it was under review by *BMC Ophthalmology*. The article was subsequently accepted and published by the *Journal of Ophthalmology* [2] and then by *BMC Ophthalmology* [1]. As it has been brought to the attention of the Editor that duplicate submission and publication have taken place, the Editor has made the decision to retract the article published in *BMC Ophthalmology* [1].

## References

[1] Demir M, Oba E, Dirim B, Ozdal E, Can E (2013). Central macular thickness in patients with type 2 diabetes mellitus without clinical retinopathy. BMC Ophthalmol.

[2] Demir M, Dirim B, Acar Z, Yilmaz M, Sendul Y (2013). Central macular thickness in patients with type 2 diabetes mellitus without clinical retinopathy. J Ophthalmol.

